# Hypoxia-Inducible Factor-1α/BNIP3-Mediated Mitochondrial Autophagy in Septic Cardiomyopathy: Protective Mechanism for Cardiac Function

**DOI:** 10.33549/physiolres.935763

**Published:** 2026-04-01

**Authors:** Qian-Qian LIU, Yu-Hong CHEN, Cong-Cong ZHAO, Zhen-Jie HU

**Affiliations:** 1 Department of Critical Care Medicine, the Fourth Hospital of Hebei Medical University, Hebei, China; 2 Department of Critical Care Medicine, the Hebei CangZhou Hospital of Integrated Traditional Chinese and Western Medicine, Hebei, China; 3 Hebei Key Laboratory of Critical Disease Mechanism and Intervention, Hebei, China

**Keywords:** BNIP3, Hypoxia-inducible factor 1α, Mitochondrial autophagy, Septic cardiomyopathy

## Abstract

This study aimed to examine the role of hypoxia-inducible factor-1α (HIF-1α) in septic cardiomyopathy (SCM), focusing on its regulatory function in mitochondrial autophagy. Differentially expressed genes (DEGs) associated with SCM were identified through analysis of the GSE79962 dataset. Mitochondrial autophagy-related genes were retrieved from the GeneCards database. Genes common to both datasets were identified using Venn diagram analysis, followed by Gene Ontology (GO) and Kyoto Encyclopedia of Genes and Genomes (KEGG) pathway enrichment analyses. A murine model of SCM was established *via* intraperitoneal injection of lipopolysaccharide (LPS). Mice were subsequently treated with either dimethyloxalylglycine (DMOG) a HIF-1α stabilizer, or 3﷓methyladenine (3-MA), an inhibitor of mitochondrial autophagy. Cardiac function, myocardial injury, inflammatory response, mitochondrial integrity, and expression levels of HIF-1α and mitochondrial autophagy markers were assessed using echocardiography, enzyme-linked immunosorbent assay (ELISA), hematoxylin–eosin staining, immunofluorescence, transmission electron microscopy, and western blot analysis. KEGG pathway analysis indicated significant enrichment of the overlapping genes in the HIF-1 signaling pathway. In vivo, DMOG administration stabilized HIF-1α expression and upregulated Bcl-2-interacting protein 3 (BNIP3), thereby enhancing mitochondrial autophagy. This enhancement was associated with reduced myocardial enzyme release and inflammatory cytokine production, as well as improvements in mitochondrial ultrastructure, myocardial histopathology, and cardiac function. In contrast, 3-MA inhibited mitochondrial autophagy and attenuated the myocardial protective effects associated with HIF﷓1α stabilization. Activation of the HIF-1α/BNIP3 signaling axis promotes mitochondrial autophagy and confers protective on cardiac function in septic cardiomyopathy. These findings present a potential mechanistic pathway and therapeutic target for mitigating myocardial injury associated with septic cardiomyopathy.

## Introduction

Sepsis is defined as life-threatening organ dysfunction resulting from a dysregulated host response to infection [1]. A substantial proportion of individuals with severe sepsis develop impaired myocardial contractility, a condition referred to as sepsis-induced myocardial dysfunction (SIMD), first described by Calvin et al. [2] This condition is also termed as sepsis-induced cardiomyopathy or septic cardiomyopathy (SC or SCM). Despite increasing recognition, diagnostic criteria remain inconsistent, and a universally accepted definition has yet to be established [3–5]. Multiple mechanisms have been proposed to contribute to the pathogenesis of SIMD, including impaired myocardial circulation, mitochondrial dysfunction, downregulation of β-adrenergic receptors, and the effects of myocardial inhibitors acting directly on cardiomyocyte receptors [6–8]. However, these mechanisms remain incompletely understood, and no targeted therapeutic strategies exist to reverse myocardial dysfunction associated with severe sepsis.

Hypoxia-inducible factor 1 (HIF-1) and their signaling pathways are integral to metabolic adaptation to hypoxic stress. HIF-1 is involved in various physiological processes, including cardiovascular development, cartilage formation, neuro-embryogenesis, and tumor progression, and is also implicated in multiple pathological processes of human diseases. Hypoxia-inducible factor 1α (HIF-1α), the oxygen-sensitive subunit of HIF-1, dimerizes with HIF-1β and translocates to the nucleus [9]. This heterodimer forms a transcriptional complex with p300, which binds to hypoxia response elements in DNA, thereby initiating the transcription of HIF-1 target genes such as vascular endothelial growth factor, glucose transporter 1, and erythropoietin [10–12].

HIF is a key transcription factor involved in cellular adaptation to variations in oxygen availability. It plays a pivotal role in metabolic regulation and is implicated in a wide range of physiological and pathological processes, including the pathogenesis of multiple diseases [13]. HIF-1α, the oxygen-sensitive subunit of the HIF complex, is one of three isoforms identified in humans: HIF-1α, HIF-2α, and HIF-3α [14,15]. Under normoxic conditions, HIF-1α undergoes rapid proteasomal degradation. However, during hypoxia, this degradation is inhibited, resulting in the stabilization and accumulation of HIF-1α protein [16]. In acute hypoxic sates, HIF-1α facilitates cellular adaptation by promoting the expression of glycolytic genes, reducing oxygen consumption, and decreasing ROS production [17].

Mitochondrial autophagy (mitophagy) refers to the selective degradation of dysfunctional or damaged mitochondria, a process essential for maintaining normal mitochondrial quality and homeostasis [18]. Bcl-2-interacting protein 3 (BNIP3), a protein localized to the mitochondrial outer membrane, plays a key role in the mitophagy regulation [19,20]. BNIP3 has been identified as a downstream target of HIF-1α, which in turn promotes BNIP3-mediated mitophagy, contributing to cellular survival during stress conditions [21].

In the present study, a combination of bioinformatics analyses and animal experiments were performed to explore the effect of HIF-1α on mitochondrial autophagy in the context of SCM. The results offer insights into the pathophysiological mechanisms underlying SCM and may support the development of effective targeted therapeutic strategies.

## Materials and Methods

### Collection and processing of datasets

**Fig. 1:**
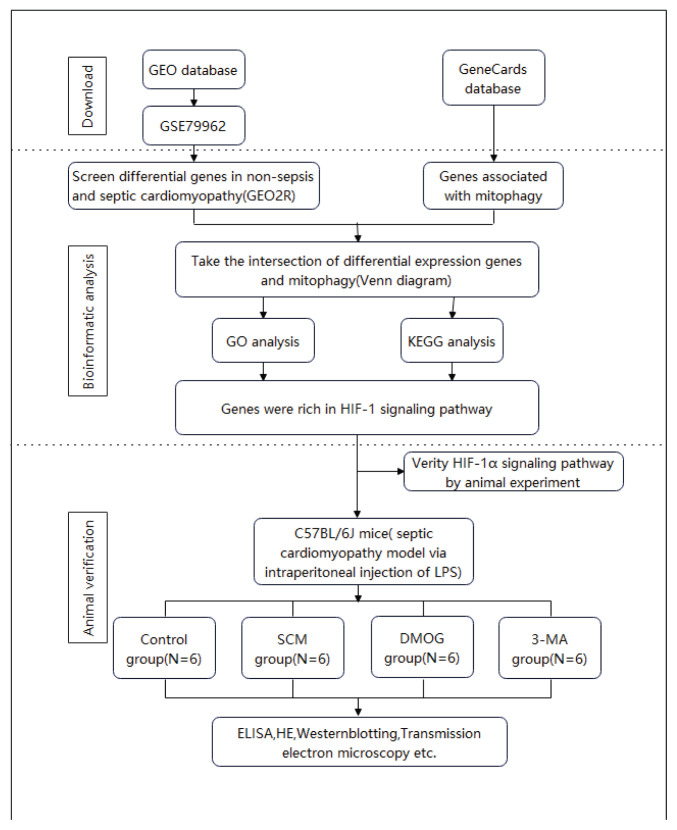
Research workflow

The gene expression dataset GSE79962, which included transcriptomic data from individuals diagnosed with SCM and non-septic controls, was obtained from the Gene Expression Omnibus (GEO) database (https://www.ncbi.nlm.nih.gov/geo/) [22]. A list of mitochondrial autophagy-associated genes was obtained from the GeneCards database (https://www.genecards.org/). The study design is illustrated in Fig. 1.

### Bioinformatics analysis

Differentially expressed genes (DEGs) were identified using the Gene Expression Omnibus 2 R (GEO2R) online tool in conjunction with the limma package in R software [23,24]. The screening criteria for DEG selection were set as |Log2FoldChange| > 1 and an adjusted *p* value Padj < 0.05. Genes overlapping between the identified DEGs and mitochondrial autophagy-associated gene set were determined using Venn diagram analysis. Gene Ontology (GO) enrichment analysis and Kyoto Encyclopedia of Genes and Genomes (KEGG) pathway enrichment analysis were conducted using the “org.Hs.eg.db” and “clusterProfiler” R software packages to identify molecular functions and key pathways associated with the overlapping genes. A Padj value of < 0.05 was applied as the threshold for selecting enriched items [25,26].

### Animals and grouping

Male C57BL/6J mice (23.5–28.5 g), aged 8–10 weeks, were obtained from Beijing HFK Bioscience Co., Ltd. and housed in an isolation room for one week to allow acclimatization. All mice were maintained under controlled environmental conditions (temperature: 20–25 °C; humidity: 50 ± 5 %), with a 12-hour light/dark cycle and ad libitum access to standard rodent chow and water. Experimental procedures were approved by the Animal Experiment Ethics Committee of the Fourth Hospital of Hebei Medical University (project license No. 2023220) and were performed in accordance with the Guidelines for the Care and Use of Laboratory Animals (National Institutes of Health, Publication No. 85-23, revised 1985). All experiments were conducted at the Animal Experiment Center of the Fourth Hospital of Hebei Medical University.

Mice were randomly assigned using the random number method into four groups (n = 6 per group): control group, SCM group, dimethyloxalylglycine (DMOG) group, and 3-methyladenine (3-MA). SCM was induced in the SCM, DMOG, and 3-MA groups by intraperitoneal injection of lipopolysaccharide (LPS; 10 mg/kg; Cat. No. L8880, Solarbio, Beijing, China) dissolved in normal saline; the control group received an equal volume of saline alone [27,28]. Cardiac function was assessed by echocardiography three hours after LPS administration [29]. Mice exhibiting evidence of myocardial dysfunction were included in subsequent analyses, whereas those without dysfunction were euthanized by cervical dislocation.

In the DMOG group, DMOG (Cat. No. 89464-63-1, GLPBIO), a HIF-1α stabilizer, was administered intraperitoneally at a dose of 50 mg/kg (dissolved in DMSO) three hours after LPS injection, in accordance with prior studies and pre-experimental validation [30,31]. In the 3-MA group, 3-MA (Cat. No. 5142-23-4, GLPBIO), a mitochondrial autophagy inhibitor, was administered intraperitoneally at a dose of 20 mg/kg (dissolved in DMSO) six hours prior to LPS administration, following established protocols [32]. Working solutions were prepared by 1:10 dilution of the DMSO stock with saline (final 10 % DMSO); each mouse received 20 μL of 10 % DMSO, equivalent to 0.1 % of body weight, a concentration well below the no-observed-adverse-effect level reported in mice [33].

Blood and myocardial tissue samples were collected six hours after LPS injection, following echocardiographic evaluation. Blood samples were centrifuged at 3000 g for 15 minutes, and plasma was stored at −80 °C. Myocardial tissue samples were also stored at −80 °C for subsequent analyses.

### Echocardiography

Mice were anesthetized using 2 % isoflurane, and ultrasound examinations were performed using a 13 MHz linear array ultrasound transducer (Vevo 2100 imaging system, VisualSonics, Ontario, Canada). All examinations were performed by a professional sonographer blinded to group allocation. M mode images were obtained in short-axis view to measure the left ventricular internal diameter at end-systole (LVIDs) and end-diastole (LVIDd). Left ventricular ejection fraction (LVEF) and left ventricular fractional shortening (LVFS) were subsequently calculated.

### Biochemical analysis

Plasma concentrations of HIF-1α (Cat. No. SEKM-0258, Solarbio, Beijing, China), cardiac troponin I (cTnI) (Cat. No. MU30257, BioSwamp, Wuhan, China), N-terminal pro-brain natriuretic peptide (NT-proBNP) (Cat. No. MU30252, BioSwamp, Wuhan, China), tumor necrosis factor-α (TNF-α) (Cat. No. MU30030, BioSwamp, Wuhan, China), and interleukin-6 (IL-6) (Cat. No. MU30044, BioSwamp, Wuhan, China) were measured using enzyme-linked immunosorbent assay (ELISA) kits, according to the instructions provided by the manufacturer.

### Histological analysis

Myocardial tissue samples were fixed in 4 % paraformaldehyde for 48 hours at room temperature, embedded in paraffin and sectioned at a thickness of 5 μm. Hematoxylin and eosin (H&E) staining was performed using reagents from Beyotime Biotech to assess myofibrillar structure and inflammatory cell infiltration. Stained sections were examined under a light microscope (DM3000 LED, Leica, Wetzlar, Germany). Myocardial necrosis and cellular infiltration were assessed by two experienced observers. For each sample, three representative sections were selected for analysis.

### Immunofluorescence

Paraffin-embedded myocardial sections were deparaffinized, subjected to antigen retrieval, and blocked with serum. Sections were incubated with a primary antibody against microtubule-associated protein light chain 3 (LC3) (1:200, AF5402, Affinity Bioscience, USA) followed by incubation with an Alexa Fluor 488-conjugated goat anti-rabbit G (IgG)-H secondary antibody (1:400, GB25303, Servicebio, Wuhan, China). Nuclei were counterstained using Beyotime c1006 (Beyotime, Nanjing, China). Immunofluorescence images were acquired using a light microscope (DM3000 LED, Leica, Wetzlar, Germany). Image analysis was subsequently conducted using ImageJ software (National Institutes of Health).

### Transmission electron microscopy

Fresh left ventricular tissue was collected, cut into approximately 1mm3 fragments, and fixed overnight at 4 °C in 2.5 % glutaraldehyde. After standard procedures for dehydration, infiltration, and embedding, ultrathin sections were prepared. Mitochondrial size, membrane structure, cristae architecture, and the presence of autophagosomes in cardiomyocytes, were examined using a Hitachi HT-7800 transmission electron microscope (Tokyo, Japan).

### Western blot analysis

Total protein was extracted from myocardial tissue by homogenization in radio-immunoprecipitation assay lysis buffer (Beyotime Biotechnology) containing protease and phosphatase inhibitors. Protein concentrations were determined using the Bicinchoninic Acid Protein Assay Kit (Cat. No. KTD3001, Abbkine Biotech, Wuhan, China). Proteins of varying molecular weights were separated by sodium dodecyl sulfate polyacrylamide gel electrophoresis (SDS-PAGE), transferred to polyvinylidene fluoride membranes, and blocked with 5 % skim milk solution. Membranes were incubated overnight with the following primary antibodies: anti-HIF-1α (1:500, AF1009, Affinity), anti-LC3A/B (1:500, AF5402, Affinity), anti-BNIP3 (1:1000, DF8188, Affinity), and anti-β-Actin (1:1000, GB15001, Servicebio). Following five washes (5 minutes each) with Tris-buffered saline containing Tween-20, membranes were incubated at room temperature for 1 hour with the appropriate horseradish peroxidase-conjugated secondary antibody. Protein bands were visualized using an enhanced chemiluminescence (ECL) detection kit (Cat. No. BMU102-CN, Abbkine, Wuhan, China), and band intensity was quantified using ImageJ software (National Institutes of Health, USA).

### Statistical analysis

Statistical analyses were conducted using SPSS version 26.0 (IBM Corp., Chicago, IL, USA) and GraphPad Prism version 9.5 (GraphPad Software Inc., San Diego, CA, USA). Data with a normal distribution were presented as mean ± standard deviation, whereas non-normally distributed data were presented as median with interquartile range (IQR). Comparisons between two groups were performed using the independent samples *t*-test, for normally distributed data and the Mann–Whitney U test for non-normally distributed data. For comparisons among multiple groups, one-way analysis of variance (ANOVA) was used for normally distributed data, while the Kruskal–Wallis test was applied for non-normally distributed data. A *p* value of < 0.05 was considered statistically significant.

## Results

### Enrichment of the HIF-1α signaling pathway in patients with SCM

**Fig. 2:**
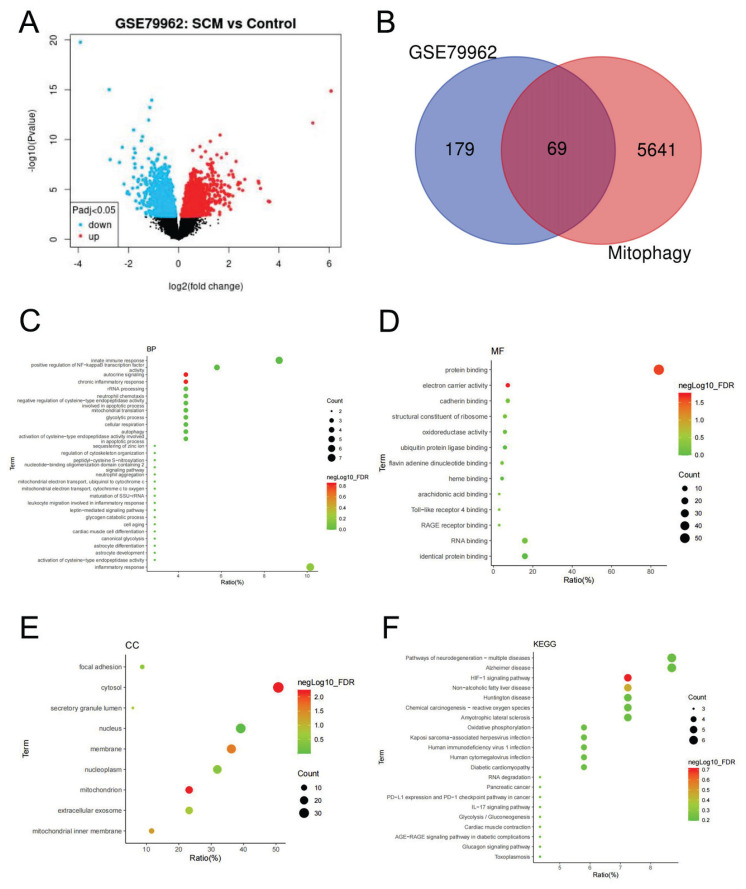
Functional and pathway enrichment analyses of overlapping genes between patients with SCM and mitochondrial autophagy-related genes. (**A**) Volcano plot of DEGs between SCM patients and non-septic patients. (**B**) Venn diagram illustrating 69 overlapping genes between DEGs and mitochondrial autophagy-related genes. (**C–E**) GO enrichment analyses of the overlapping genes. (**F**) KEGG pathway enrichment analysis of the overlapping genes.

Analysis of the GSE79962 dataset indicated 248 DEGs in myocardial tissue from patients with SCM compared with non-septic controls, including 155 upregulated and 93 downregulated genes (Fig. 2A). A total of 5,710 mitochondrial autophagy-related genes were identified from the GeneCards database. Venn diagram analysis identified 69 overlapping genes between the DEGs and the mitochondrial autophagy-related gene set (Fig. 2B). GO molecular function enrichment analysis indicated that these overlapping genes were associated with autocrine signaling, chronic inflammatory response, protein binding, electron carrier activity, cytoplasmic localization, and mitochondrial components (Fig. 2C–E). KEGG pathway enrichment analysis demonstrated significant enrichment of these genes in the HIF-1α signaling pathway (Fig. 2F).

### Role of HIF-1α in a murine model of LPS-induced SCM

**Fig. 3:**
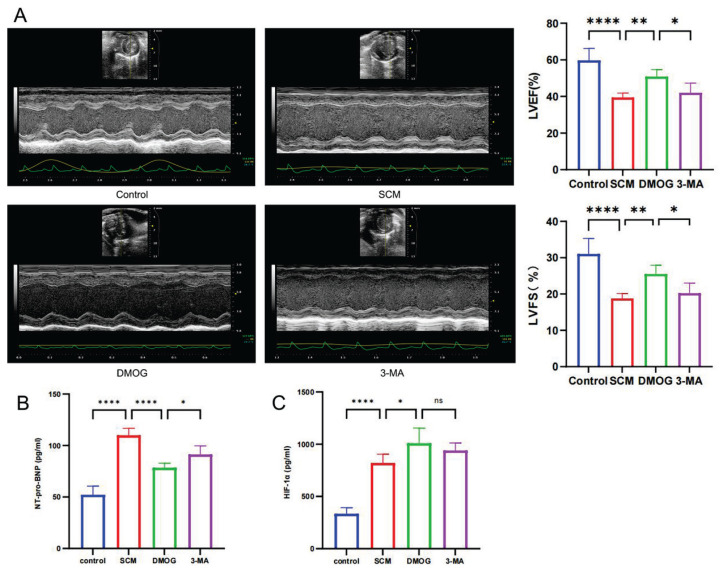
HIF-1α improves cardiac function LPS-induced SCM. (**A**) Representative M-mode echocardiographic images and quantitative analysis of LVEF and LVFS (n = 6 per group) (**B**) Plasma NT-proBNP levels for cardiac function assessment (n = 6 per group); (**C**) Plasma HIF-1α levels in each group (n = 6 per group). Statistical significance: * *p* < 0.05, ** *p* < 0.01, *** *p* < 0.001, **** *p* < 0.0001.

No significant differences in body weight were observed among the groups, and no mortality occurred during the experiment. Six hours after intraperitoneal LPS injection, mice exhibited signs of endotoxemia, including lethargy, reduced activity, generalized piloerection, diarrhea, and increased ocular secretions. Echocardiographic evaluations demonstrated a significant reduction in LVEF and LVFS in the SCM group compared with the control group (Fig. 3A). These changes were accompanied by a significant increase in peripheral blood NT-proBNP levels (Fig. 3B). Myocardial HIF-1α expression was upregulated after LPS exposure (Fig. 3C). In the DMOG group, treatment with DMOG, a HIF-1α stabilizer, further increased HIF-1α expression compared with the SCM group (Fig. 3C) and significantly improved LVEF, LVFS, and NT-proBNP levels (Figs 3A, B). In contrast, treatment with 3-MA, a mitochondrial autophagy inhibitor, did not significantly alter HIF-1α expression compared with the DMOG group (Fig. 3C), but was associated with increased NT-proBNP levels and decreased LVEF and LVFS (Fig. 3A, B).

### Role of HIF-1α in cardiac inflammation, cardiomyocyte injury, and myocardial pathology in mice with LPS-induced SCM

**Fig. 4:**
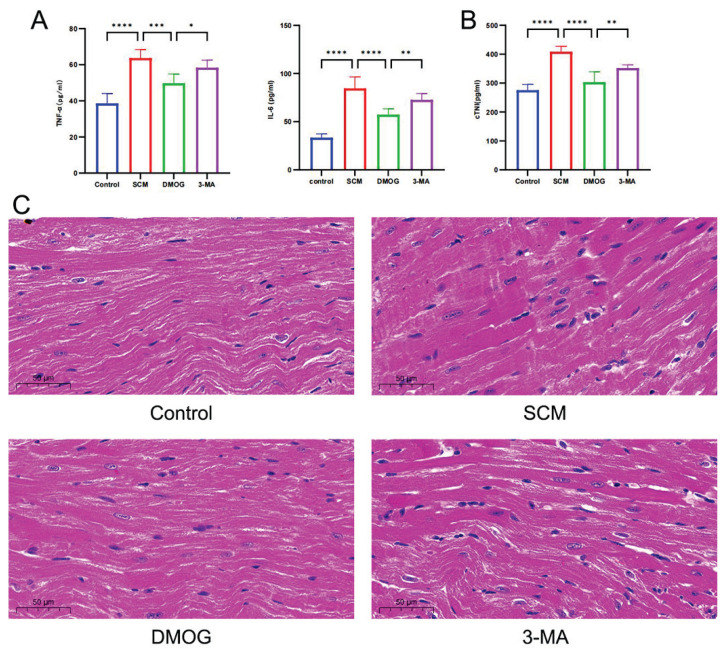
HIF-1α reduces cardiac inflammation, cardiomyocyte injury, and histopathological damage. (**A**) Plasma levels of TNF-α and IL-6 as markers of inflammation (n = 6 per group); (**B**) Plasma cTnI levels as a marker of myocardial injury (n = 6 per group); (**C**) Repre-sentative H&E-stained myocar-dial tissue sections (n = 6 per group). Statistical significance: * *p* < 0.05, ** *p* < 0.01, *** *p* < 0.001, **** *p* < 0.0001

In the SCM group, plasma levels of TNF-α and IL-6 were significantly elevated compared with the control group. Pharmacological stabilization of HIF-1α using DMOG significantly reduced TNF-α and IL-6 levels, whereas inhibition of mitochondrial autophagy with 3-MA resulted in a significant increase in TNF-α and IL-6 levels compared with the DMOG group (Fig. 4A). A similar trend was observed for cardiac troponin (cTnI), a biomarker for the cardiomyocyte injury (Fig. 4B).

Histopathological examination of H&E stained myocardial sections indicated prominent cardiomyocyte edema, disrupted cellular arrangement, and significant inflammatory cell infiltration in the SCM group compared with the control group. These pathological changes were significantly alleviated in the DMOG group following HIF-1α stabilization. In contrast, administration of 3-MA aggravated cardiomyocyte injury compared to the DMOG group, consistent with the inhibition of mitochondrial autophagy (Fig. 4C).

### Role of HIF-1α in mitochondrial injury in cardiomyocytes of mice with LPS-induced SCM

**Fig. 5:**
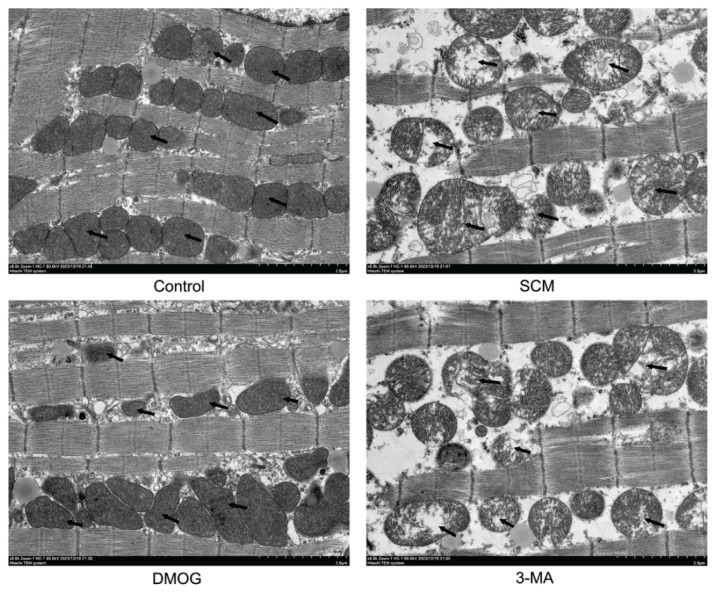
HIF-1α mitigates mitochondrial damage in cardio-myocytes. Repre-sentative TEM images showing mitochondrial ultrastructure in each group (n = 6 per group).

Transmission electron microscopy (TEM) indicated well-preserved mitochondrial ultrastructure in the control group, characterized by intact membranes, neatly arranged cristae, uniform matrix density, and orderly mitochondrial arrangement. In the SCM group, mitochondria displayed significant swelling, disrupted arrangement, membrane blurring and damage, fragmented and diminished cristae, and reduced, faded matrix density. In the DMOG group, mitochondrial swelling was notably reduced, arrangement was relatively orderly, membranes were predominantly intact, cristae were neatly arranged, and matrix density was uniform. In the 3-MA group, mitochondria exhibited swelling, membrane damage, broken cristae, and decreased, faded matrix density (Fig. 5).

### HIF-1α protects cardiac function in mice with LPS-induced SCM through modulation of mitochondrial autophagy

**Fig. 6:**
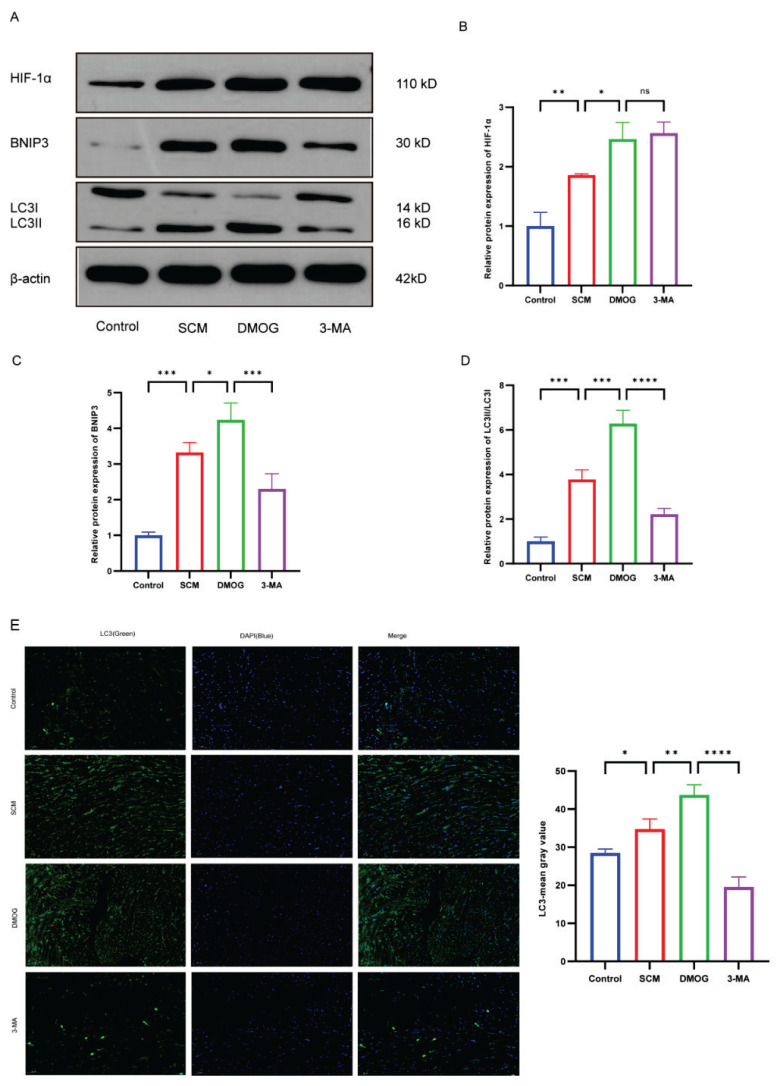
Effect of HIF-1α on BNIP3-mediated mitochondrial autophagy in myocardial tissue. (**A**) Repre-sentative western blot images from each group; (**B–D**) Quantitative analysis of HIF-1α expression, BNIP3 expression, and the LC3B-II/ LC3B-I ratio in myocardial tissue from each group (n = 6 per group). (**E**) Representative immunofluo-rescence images showing LC3 expression in left ventricular tissue from each group (n = 6 per group); LC3-positive staining is shown in green and nuclei are counterstained with DAPI (blue). Quantitative analysis of LC3-positive signal is presented for each group (n = 6 per group). Statistical significance: *p<0.05, **p<0.01, ***p<0.001, ****p<0.0001, ns indicates not significant.

Western blot analysis of myocardial tissue demonstrated that intraperitoneal injection of LPS in mice led to increased HIF-1α expression, as well as upregulation of BNIP3 and LC3. Administration of DMOG stabilized HIF-1α, further increasing its protein expression and enhancing BNIP3 and LC3 expression. In contrast, treatment with 3-MA significantly suppressed BNIP3 and LC3 expression, while HIF-1α levels remained unchanged (Fig. 6A–D).

To further examine the relationship between HIF-1α and mitochondrial autophagy, immunofluorescence staining was performed. DMOG treatment resulted in an increase in LC3-positive punctate staining, consistent with the induction of mitochondrial autophagy. Conversely, 3-MA treatment reduced LC3-positive puncta, indicating suppression of autophagosome formation (Fig. 6E).

## Discussion

Sepsis is a systemic inflammatory condition resulting from a dysregulated host response to infection, often progressing to life-threatening multi-organ dysfunction. SCM is a common but potentially reversible complication of sepsis that has received increasing attention in recent years due to its association with high morbidity and mortality rates [34].

In the present study, bioinformatics analysis was used to identify DEGs between individuals with SCM and non-septic controls. A subset of genes associated with both SCM and mitochondrial autophagy was determined using Venn diagram analysis. KEGG pathway enrichment analysis indicated that these overlapping genes were enriched in the HIF-1α signaling pathway. Within this pathway BNIP3 was identified as a key downstream effector of HIF-1α involved in the regulation of mitochondrial autophagy.

To validate these findings and explore the role of the HIF-1α/BNIP3 axis in SCM, an LPS-induced murine model of SCM was employed. The results demonstrated that, compared with the control group, peripheral blood levels of cTnI, NT-proBNP, and inflammatory markers such as TNF-α and IL-6 were significantly elevated in mice with SCM. Stabilization of HIF-1α using DMOG resulted in a reduction in these biomarkers. Concurrently, increased HIF-1α expression was associated with a significant upregulation of BNIP3 and an elevated LC3II/LC3I ratio in myocardial tissue, indicating activation of mitochondrial autophagy.

Western blot analysis confirmed that the expression levels of HIF-1α and BNIP3 were significantly elevated in myocardial tissue from SCM mice. DMOG treatment further increased HIF-1α expression and was associated with enhanced BNIP3 expression and a higher LC3II/LC3I ratio. These molecular changes were accompanied by improved cardiac function reflected by echocardiographic parameters and reduced levels of NT-proBNP. In contrast, inhibition of mitochondrial autophagy using 3-MA suppressed BNIP3 LC3 expression without affecting HIF-1α levels. This suppression was associated with worsened cardiac function.

Previous studies have indicated that HIF-1α plays an important role in preserving cardiac function [35–37]. It has been reported that HIF-1α attenuates myocardial inflammatory injury induced by coronary microembolism via inhibition of the Toll-like receptor (TLR4)/myeloid differentiation primary response 88 (MyD88/nuclear factor κB (NF-κB)) signaling pathway, thereby improving cardiac performance [38]. HIF-1α has been reported to enhance myocardial tolerance to ischemic and hypoxic injury [9]. Furthermore, ubiquitin carboxy-terminal hydrolase L1 protects cardiac function following myocardial infarction by stabilizing HIF-1α and activating the HIF-1α signaling pathway [39]. Naringin, a flavonoid compound, has been shown to protect H9C2 cardiomyoblasts from hypoxia-induced injury by enhancing autophagy through activation of the HIF-1α/BNIP3 signaling axis [40].

In this study, elevated expression of HIF-1α was associated with reduced peripheral blood levels of cTnI and NT-proBNP, attenuation of myocardial histopathological damage, and preservation of mitochondrial ultrastructure in mice with LPS-induced SCM. These changes were accompanied by improvements in cardiac function, as evidenced by increased LVEF and LVFS on echocardiographic assessment, findings that align with those reported by Yu *et al.* who demonstrated a cardioprotective role of HIF-1α in septic conditions [41]. In contrast, Pan et al. reported that naringenin may exert a protective effect against LPS-induced cardiomyocyte injury by binding directly to HIF-1α and inhibiting the release of inflammatory cytokines [42].

The differences between the results of the two studies may be attributable to several factors. First, differences in the timing of tissue collection may have influenced the observed outcomes. Although both studies used a similar model of SCM induced by intraperitoneal injection of LPS (10 mg/kg), the current study performed assessments six hours post-injection, while Pan *et al.* evaluated outcomes at twelve hours. The current findings may therefore reflect the effects of HIF-1α in the early phase of SCM. Whether the role of HIF-1α in cardiac function changes over the course of sepsis progression remains unclear and warrants further investigation. Second, the two studies examined distinct biological mechanisms: Pan et al. focused primarily on the regulation of inflammatory cytokine release, whereas the present study focused on HIF-1α–mediated mitochondrial autophagy. Given that SCM is characterized by ischemia and hypoxia in cardiomyocytes, it is plausible that multiple signaling pathways are activated to mitigate tissue injury, many of which are regulated by HIF-1α. However, the role of the HIF-1α-mediated hypoxia signaling in the context of SCM has not been extensively explored. Further mechanistic studies are needed to clarify the precise functions of HIF-1α in the pathophysiology of septic cardiomyopathy.

Mitochondrial autophagy is a cellular protective mechanism that functions an acute response to tissue stress, facilitating the clearance of excess or dysfunctional mitochondria and thereby maintaining mitochondrial network integrity [43]. Impairment of mitochondrial autophagy has been implicated in the pathogenesis of various conditions, including neurodegenerative diseases, heart failure, cancer, and age-related decline in cellular function [44]. Lin *et al.* reported that roxadustat, a HIF prolyl hydroxylase inhibitor, protects tubular epithelial cells from iohexol-induced injury in vivo and in vitro by stabilizing HIF-1α and activating downstream BNIP3-mediated mitochondrial autophagy. Notably, deficiency of BNIP3 markedly reduced mitochondrial autophagy and exacerbated cellular apoptosis and renal injury [45]. Similarly, Zhang et al. demonstrated that BNIP3 confers protection against cardiomyocyte ischemia–reperfusion injury in vivo and in vitro by promoting mitochondrial autophagy [46]. Han et al. further indicated that inhibition of the mechanistic target of rapamycin (mTOR) pathway, through the acceleration of autophagy, exerts a cardioprotective effect in septic-induced myocardial dysfunction (SIMD) [47].

In this study, intraperitoneal injection of LPS in mice resulted in increased inflammatory markers, elevated myocardial injury indicators in peripheral blood, and impaired cardiac function. Histopathological examination indicated inflammatory cell infiltration and myocardial structural injury, while TEM demonstrated disruption of mitochondrial ultrastructure in cardiomyocytes. These changes were accompanied by reductions in LVEF and LVFS on echocardiography, consistent with septic cardiomyopathy–associated cardiac dysfunction.

Treatment with DMOG, a prolyl hydroxylase inhibitor, stabilized HIF-1α expression in myocardial tissue, which in turn activated the mitochondrial autophagy receptor BNIP3. This promoted mitochondrial autophagy, facilitating the clearance of dysfunctional mitochondria and reducing the cytoplasmic release of mitochondrial DNA, ROS, nitric oxide, and cytochrome c—key mediators of oxidative stress and apoptosis. Consequently, mitochondrial injury was attenuated, cardiomyocyte damage was mitigated, and cardiac function was improved. These findings align with the report by Yu et al., which demonstrated that BNIP3 upregulation activates autophagy, reduces apoptosis, and confers cardioprotection in SIMD [42].

To confirm that HIF-1α mediates its effects *via* the regulation of mitochondrial autophagy, 3-MA, an autophagy inhibitor, was administered. Although HIF-1α expression in the cardiac tissue remained comparable to that in the DMOG-treated group, inhibition of BNIP3 suppressed mitochondrial autophagy. This suppression was associated with aggravated cardiomyocyte injury and mitochondrial function, as evidenced by elevated levels of cTnI and NT-proBNP, worsened myocardial histopathology, mitochondrial damage observed on TEM, and further reductions in LVEF and LVFS. These findings indicate that HIF-1α may improve cardiac function in LPS-induced SCM, at least in part, through the promotion of BNIP3-dependent mitochondrial autophagy.

However, the current understanding of the HIF-1α/BNIP3 signaling axis in the context of SCM remains limited. Further mechanistic studies are warranted to elucidate the precise role of this pathway and to explore its potential as a therapeutic target in sepsis-related cardiac dysfunction.

This study has several limitations that should be acknowledged. First, although the LPS-induced murine model of septic cardiomyopathy (SCM) recapitulates key aspects of the human condition, important differences remain. In particular, the etiology of SCM in humans involves a broader range of pathogens, including both Gram-negative and Gram-positive bacteria, whereas the experimental model employed here involved a single Gram-negative endotoxin. Second, the study did not include the use of HIF-1α knockout mice, which limits the ability to definitively confirm the specific role of HIF-1α in the observed cardioprotective effects. Third, the investigation was limited to in vivo experiments, The absence of complementary in vitro studies in cardiomyocytes or relevant cell lines precludes further mechanistic validation of the HIF-1α/BNIP3 signaling pathway and its direct impact on mitochondrial autophagy.

## Conclusion

In summary, this study identified a potential association between the HIF-1α/BNIP3 signaling pathway and septic cardiomyopathy through bioinformatics analysis and in vivo validation. The findings suggest that activation of the HIF-1α/BNIP3 axis confers cardioprotective effects by promoting mitochondrial autophagy, thereby mitigating mitochondrial injury and improving cardiac function. These results offer new insights into the pathophysiological mechanisms underlying SCM and highlight a potential therapeutic target for future intervention strategies.

## Abbreviations

HIF-1α: hypoxia-inducible factor 1α
BNIP3: Bcl-2 interacting protein 3
GEO: Gene Expression Omnibus
GO: Gene Ontology
KEGG: Kyoto Encyclopedia of Genes and Genomes
DMOG: dimethyloxalylglycine
3-MA: 3-methyladenine
ELISA: Enzyme-Linked Immunosorbent Assay
GSE: Gene Expression Series
SIMD: sepsis-induced myocardial dysfunction
SIC: sepsis-induced cardiomyopathy
SC or SCM: septic cardiomyopathy
HRE-DNA: hypoxia response element DNA
VEGF: vascular endothelial growth factor
GLUT1: glucose transporter 1
EPO: erythropoietin
GEO2R: Gene Expression Omnibus 2 R
DEGs: differentially expressed genes
LPS: lipopolysaccharide
DMSO: Dimethyl Sulfoxide
LVIDs: left ventricular internal diameter at end-systole
LVIDd: left ventricular internal diameter at end-diastolic
LVEF: left ventricular ejection fraction
LVFS: left ventricular fractional shortening
cTnI: cardiac troponin I
NT-proBNP: N-terminal pro-brain natriuretic peptide
TNF-α: tumor necrosis factor α
IL-6: interleukin-6
LC3: light chain 3
RIPA: radio-immunoprecipitation assay
BCA: Bicinchoninic Acid
SDS-PAGE: Sodium Dodecyl Sulfate PolyAcrylamide Gel Electrophoresis
PVDF: Polyvinylidene Fluoride
TBST: Tris-Buffered Saline with Tween
ECL: Enhanced chemiluminescence
ANOVA: one-way analysis of variance
M: mitochondria
HE: hematoxylin-eosin
WB: Western blot
ROC: reactive oxygen species
TLR4: Toll-like receptor 4
MyD88: myeloid differentiation primary response 88
NF-κB: nuclear factor κ-B
UCHL1: Ubiquitin carboxy-terminal hydrolase L1
mTOR: mechanistic target of Rapamycin
mtDNA: mitochondrial DNA
NO: nitric oxide
